# Horizontal Heat Impact of Urban Structures on the Surface Soil Layer and Its Diurnal Patterns under Different Micrometeorological Conditions

**DOI:** 10.1038/srep18790

**Published:** 2016-01-05

**Authors:** Hongxuan Zhou, Dan Hu, Xiaolin Wang, Fengsen Han, Yuanzheng Li, Xiaogang Wu, Shengli Ma

**Affiliations:** 1 State Key Laboratory of Urban and Regional Ecology, Research Center for Eco-Environmental Sciences, Chinese Academy of Sciences, Beijing 100085, PR China; 2 College of Urban and Rural Construction, Shanxi Agricultural University, Taigu County, Shanxi Province, 030801, PR China; 3 Environment and Sustainability Institute, University of Exeter, Penryn, Cornwall TR10 9FE, UK; 4 College of Eco-Environmental Engineering, Qinghai University, Xining, Qinghai, 810016, PR China

## Abstract

The temperature of the surface soil layer around different orientation walls was investigated horizontally along several construction-soil micro-gradients in Beijing, China. On a diurnal scale, similar fluctuating trends in T_0_ and T_50_ (temperature of surface soil layer, 0 and 0.5 m from the building baseline) adjacent to the external walls of buildings with the same orientation usually appeared under similar micrometeorological conditions. The difference between T_0_ and T_50_ (ΔT_0–50_) can be considered an indicator of the intensity of the horizontal heat effects: higher ΔT_0–50_ values correspond to greater intensities. The values of ΔT_0–50_ for south-, north-, east- and west-facing sides of buildings were highest on sunny days in summer and exhibited values of 6.61 K, 1.64 K, 5.93 K and 2.76 K, respectively. The scope of horizontal heat impacts (S_h_) changed on a diurnal scale between zero and the maximum, which fluctuated with the micrometeorological conditions. The maximum values of S_h_ were 0.30, 0.15, 0.20 and 0.20 m for south-, north-, east-, and west-facing walls. The ΔT_0–50_ was related to solar radiation, horizontal heat flux, relative humidity, wind speed, soil moisture differences and air temperature; the relative importance of these factors was 36.22%, 31.80%, 19.19%, 2.67%, 3.68% and 6.44%, respectively.

In recent decades, intensive regional urbanization has significantly changed the land surface properties. Large quantities of building materials, such as concrete and asphalt, have been widely used and have replaced the original vegetation cover^1^. Consequently, the albedo of the land surface, heat capacity of the soil, transpiration efficiency and surface roughness of the land cover have changed greatly in urban areas, leading to higher atmospheric temperatures in urban areas than in rural or natural regions, a phenomenon known as the urban heat island (UHI)^2^,^3^. Due to the long-term impacts on atmospheric processes, weather conditions, functioning of urban green spaces and human health, the UHI effect has become a great concern in recent years^4^,^5^,^6^,^7^. Previous studies have shown that the maximum intensity of the heat island effect was 8 K in New York City, that the air temperature is 5–10 K higher in downtown areas of Baltimore than that in rural areas, and that Tokyo’s air temperatures have risen faster than other cities around the world, increasing by 3 K in the past 100 years, i.e., 5 times faster than global warming^1^,^8^,^9^. In addition to atmospheric temperatures, urban soil temperatures are also rising due to the UHI effect. Soil temperatures exhibit a significant, highly positive correlation with air temperature. Many researchers have reported an upward trend in soil temperatures and even ground water temperature in urban areas^10^,^11^,^12^,^13^,^14^,^15^,^16^.

Heat transfer is another driving force in the changes in the distribution of soil temperatures within urban areas, except for the shading of buildings. Many of the previous studies on heat transfer in soil have focused on improving energy conservation and less on the soil thermal environment. Using Philip and De Vries’ classic theory and combining the properties of soil and building materials, Santos and Mendes^17^ reported that soil temperature and water content affected heat processes in low-rise buildings. Givoni^18^ showed that soil could become a cold source for adjacent buildings to keep them from reaching excessively high temperatures in Saudi Arabia and other hot regions. Mihalakakou^19^ revealed the regularity of soil temperature changes beneath structures in an external environment with constantly fluctuating temperatures by establishing an accurate model to predict the changes in soil temperature beneath structures. Landman and Delsante^20^ calculated the quantitative heat transport between the soil and insulating the insulating layers of building walls made of different materials. Menberg^21^ and Benz^22^ noted that heat flux from infrastructures was the main driving force to increase the temperature of shallow aquifers in urban areas. These previous studies have confirmed that heat transfer occurs between buildings and the soil and that the buildings act as the heat source. Similarly, roads also function as heat sources that transport heat to the soil. Dambros^23^ stated that roads have higher temperatures than their surrounding soil environments and are harmful to the adjacent vegetated land. Yang^24^ reported higher temperatures in the soil next to roads without analysing in detail how the roads exerted a horizontal impact on the soil temperature. Delgado^25^ found that roads are heat sources on islands and maintain relatively higher temperatures than those of the soil in the surrounding forest. The combined effects of both higher road temperatures and the canopy density of the forest led to a decreasing trend in the first three metres of the ecotone between the roads and the forest. Soil temperatures are also directly linked to many ecosystem processes, such as soil heterotrophic respiration, microbial decomposition, nutrient cycling, and root respiration, which are influenced by soil temperature^26^,^27^, which suggested that adjacent structures in urban areas affected these processes. Consequently, the heat impact process between structures and soil is a critical element in the regulation of these ecological processes. Therefore, exploring the heat effects of structures on soil is of great scientific significance.

This study aims to contribute to the understanding of the horizontal heat impacts of urban buildings on soil surface layer temperatures in the adjacent green space across different micrometeorological conditions created by the urban buildings themselves. A range of ecotones between the urban structures and the soil were selected as *in situ* observation sites, in which the temperature of the surface soil layer (soil temperature) was observed and logged to explore the horizontal heat impacts on soil temperature caused by various external walls of buildings (external wall), which yielded different micrometeorological conditions. This study focused on the diurnal pattern of horizontal heat impacts of urban structures on the temperature of the soil in green spaces adjacent to the buildings under various micrometeorological conditions in different weather situations and seasons.

## Results

### Hourly changes in the soil temperature and the intensity of horizontal heat impact on a diurnal scale

**Figure 1:**
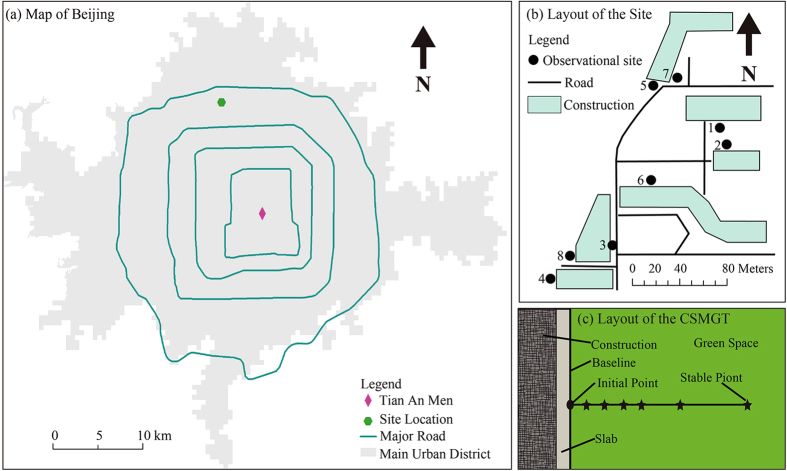
Locations of the observation sites for the CSMGT. (**a**) *Map of Beijing* and (**b**) *Layout of the site* were created with the ESRI ArcGis9.3 software and modified with the Adobe Illustrator CS4 software. (**c**) *Layout of the CSMGT* was created with the Adobe Illustrator CS4 software.

Horizontally, the temperature of a surface soil layer (hereafter called soil temperature) was investigated along the construction-soil micro-gradient transect (referred to as the CSMGT henceforth, see the Methods section). The soil temperatures of the Initial Point (0 m from baseline) and the Stable Point (0.5 m from baseline) and the intensity of the horizontal heat impacts (difference between the temperatures of the Initial Point and the Stable Point) were recorded as T_0_, T_50_ and ΔT_0–50_ respectively. The *in situ* observations were performed on a 24-hour basis; a daily cycle started at 6:00 (approximately sunrise) and ended at 5:59 the next day. The layout of the site is shown in Fig. 1.

**Figure 2:**
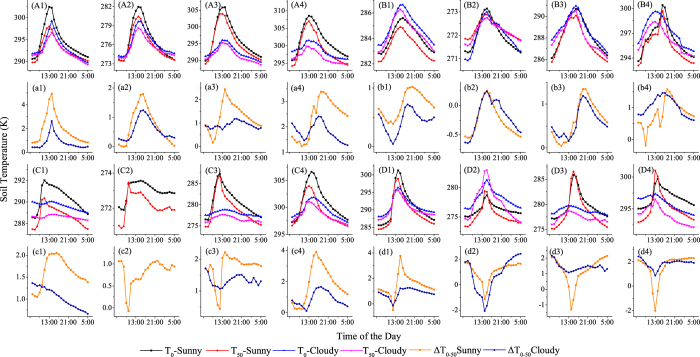
T_0_, T_50_ and ΔT_i-50_ of green space soil adjacent to different external walls under various weather conditions and seasons. (**A1**) and (**a1**): South-facing wall, autumn, site 1; (**A2**) and (**a2**): South-facing wall, winter, site 5; (**A3**) and (**a3**): South-facing wall, spring, site 1; (**A4**) and (**a4**): South-facing wall, summer, site 1; (**B1**) and (**b1**): North-facing wall, autumn, site 2; (**B2**) and (**b2**): North-facing wall, winter, site 2; (**B3**) and (**b3**): North-facing wall, spring, site 6; (**B4**) and (**b4**): North-facing wall, summer, site 2; (**C1**) and (**c1**): East-facing wall, autumn, site 3; (**C2**) and (**c2**): East-facing wall, winter, site 3; (**C3**) and (**c3**): East-facing wall, spring, site 3; (**C4**) and (**c4**): East-facing wall, summer, site 7; (**D1**) and (**d1**): West-facing wall, autumn, site 4; (**D2**) and (**d2**): West-facing wall, winter, site 8; (**D3**) and (**d3**): West-facing wall, spring, site 8; (**D4**) and (**d4**): West-facing wall, summer, site 4.

Figure 2 shows T_0_, T_50_ and ΔT_0–50_ adjacent to different external walls of buildings under different weather conditions and seasons. The higher values of ΔT_0–50_ clearly correlate with higher horizontal heat impact intensities.

Table 1 shows the dynamic situation in detail under different weather conditions and seasons, including the maximum values, minimum values and the timing of their occurrence for T_0_, T_50_ and ΔT_0–50_ on a diurnal scale. Table 1 also lists the duration of time during which buildings acted as heat sources. Due to the non-existence of the situation in which T_0_ was equal to T_50_, heat flow was always present between these two points.

In general, the changes in T_0_ and T_50_ always shared a similar periodic curve at different external walls in different weather and seasons. For south-facing external walls, T_0_ and T_50_ were highest during 12:00–15:00 (one hour earlier or later, depending on the weather conditions); for north-facing external walls, the maximum values of T_0_ and T_50_ appeared in the afternoon, between 13:00–16:00; for the east-facing external walls, both maximum values appeared between 11:00 and 15:00 (depending on the weather conditions); and for west-facing external walls, the temperatures were usually highest during 13:00–15:00. The minimum values of T_0_ and T_50_ for the four different external walls occurred at dawn or morning and were subject to the weather conditions and the duration of sunshine. The difference between T_0_ and T_50_ (ΔT_0–50_) also exhibited a curve of diurnal fluctuations, and ΔT_0–50_ was considered an effective indicator of the intensity of the horizontal heat impact. The maximum ΔT_0–50_ values appeared on sunny days in summer: 6.61 K, 1.64 K, 5.93 K and 2.76 K for the south-, north-, east- and west-facing sides of buildings, respectively. The greatest intensity of horizontal heat impacts (ΔT_0–50_) of a building appeared on the south-facing side on sunny days in summer.

### Per hour changes in the scope of horizontal heat impact on a diurnal scale

**Figure 3:**
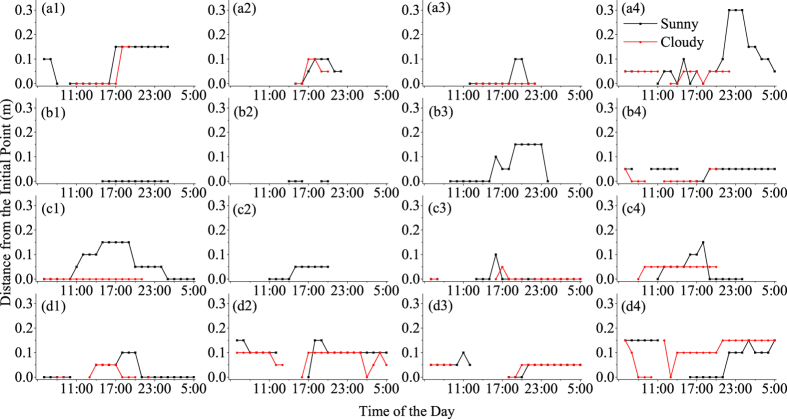
Changes in the scope of horizontal heat impacts on soil in green space adjacent to different external walls in different weather conditions and seasons. (**a1**): South-facing wall, autumn, site 1; (**a2**): South-facing wall, winter, site 5; (**a3**): South-facing wall, spring, site 1; (**a4**): South-facing wall, summer, site 1; (**b1**): North-facing wall, autumn, site 2; (**b2**): North-facing wall, winter, site 2; (**b3**): North-facing wall, spring, site 6; (**b4**): North-facing wall, summer, site 2; (**c1**): East-facing wall, autumn, site 3; (**c2**): East-facing wall, winter, site 3; (**c3**): East-facing wall, spring, site 3; (**c4**): East-facing wall, summer, site 7; (**d1**): West-facing wall, autumn, site 4; (**d2**): West-facing wall, winter, site 8; (**d3**): West-facing wall, spring, site 8; (**d4**): West-facing wall, summer, site 4.

Hourly changes in the scope of horizontal heat impacts (S_h_) on the soil under different micrometeorological conditions are shown in Fig. 3. The temporal unit of observation was set to exactly 24 hours, from 6:00 (approximately sunrise) to 5:59 the next day.

Table 2 shows the maximum values of S_h_ (S_h-max_) and the total temporal duration of impacts (Dur_total_) for different external walls in different weather conditions and seasons. The maximum values of S_h_ are usually larger on sunny days than on cloudy days in the same season, except for the south-facing external wall in winter and the north-facing external wall in summer. The maximum values of S_h_ (S_h-max_) differ between different the external walls of buildings within the same seasons, whereas the minimum values of S_h_ do not exist at all. In addition, the values of S_h-max_ differ between seasons, and larger values of S_h_ are present in summer days than in winter days.

According to Table 2, the values of Dur_total_ vary with changing weather and seasons, and these values are usually larger on sunny days than on cloudy days, except for the south-facing external wall in winter and the north-facing external wall in summer. However, for the west-facing external wall, the value of Dur_total_ is larger on cloudy days than on sunny days in summer and winter.

Along the CSMGT, the maximum scope of horizontal heat impacts appeared after nightfall when solar radiation was weak or absent. The south-, north-, east- and west-facing sides of buildings had maximum horizontal heat impacts values at 0.30 m, 0.15 m, 0.20 m and 0.20 m, respectively. The horizontal heat impacts exhibited a similar pattern of cyclic and periodic changes that were obviously influenced by weather and seasonal conditions.

### Distribution of the mean soil temperature along the CSMGT

The soil temperature of green space adjacent to different external walls was investigated continuously in every season for periods of 4–18 days, depending on the weather and meteorological conditions, including sunny and cloudy days. Table 3 lists the temporal durations of the observations in different seasons. Based on the continuous investigations, the mean soil temperature for every observation point in the CSMGT was order to identify the diurnal dynamics of the soil temperature in structure-adjacent green spaces in different seasons.

The mean soil temperature is defined as the average soil temperature of each observation point during an independent period of observation in the same weather conditions and seasons.


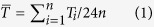


where 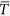 is the mean soil temperature, T_i_ is the soil temperature of each observation point, and n is the total number of observation days in an independent period of observation.

**Figure 4:**
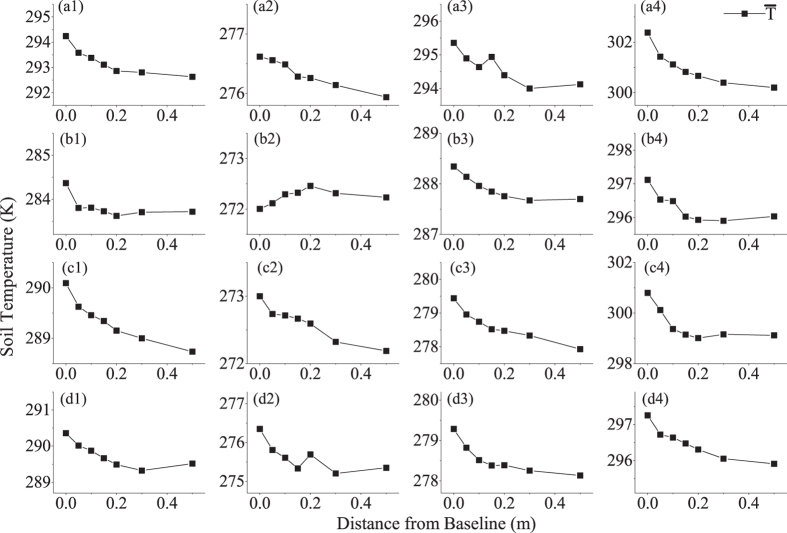
Distribution of mean soil temperatures adjacent to different external walls in different seasons. (**a1**): South-facing wall, autumn, site 1; (**a2**): South-facing wall, winter, site 5; (**a3**): South-facing wall, spring, site 1; (**a4**): South-facing wall, summer, site 1; (**b1**): North-facing wall, autumn, site 2; (**b2**): North-facing wall, winter, site 2; (**b3**): North-facing wall, spring, site 6; (**b4**): North-facing wall, summer, site 2; (**c1**): East-facing wall, autumn, site 3; (**c2**): East-facing wall, winter, site 3; (**c3**): East-facing wall, spring, site 3; (**c4**): East-facing wall, summer, site 7; (**d1**): West-facing wall, autumn, site 4; (**d2**): West-facing wall, winter, site 8; (**d3**): West-facing wall, spring, site 8; (**d4**): West-facing wall, summer, site 4.

The distribution of mean soil temperatures is shown in Fig. 4, which includes the data for different external walls in different seasons.

Table 3 lists the differences in the mean soil temperature as ΔMT_0–50_ between the Initial Point and the Stable Point; this variable can be used to express the intensity of the average building-induced horizontal heat impacts in the CSMGT. In addition the table lists the direction of heat flow, using an arrow symbol for different seasons.

The distribution of the mean soil temperature for different seasons along the CSMGT showed a similar decreasing trend, except for the north-facing external wall in winter. The mean soil temperature was higher for south-facing external walls in summer than any other external wall in any other season. The average value of ΔT_0–50_ was 2.18 K for the south-facing external wall in summer and −0.23 K for the north-facing external wall in winter.

### Stepwise regression results

According to the method that was adopted in previous research^28^,^29^, stepwise regression was performed. The stepwise regression results are presented in Table 4, where the unstandardized coefficient b is the multiplicative factor of each parameter in the regression model, whereas the standardized coefficient Beta is the indicator of the relative importance among the parameters (the greater the absolute value is, the higher the relative importance is). The term Sig represents the statistical significance of the results.

**Figure 5:**
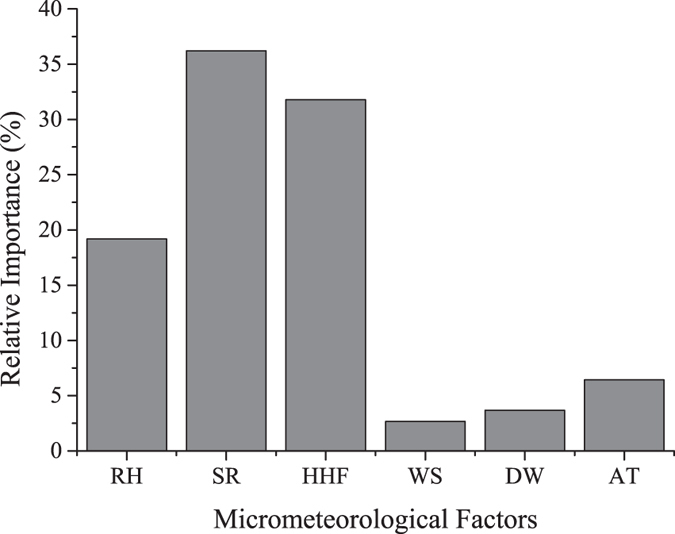
Relative importance of the meteorological factors. RH is relative humidity, SR is solar radiation, HHF is horizontal heat flux, WS is wind speed, DW is the difference in soil moisture, and AT is air temperature.

According to Fig. 5, different meteorological factors had different relative importance values. Relative humidity, solar radiation, horizontal heat flux, wind speed, soil moisture and air temperature featured values of 19.19%, 36.22%, 31.80%, 2.67%, 3.68% and 6.44%, respectively.

## Discussion

### Patterns of the horizontal heat impact cycle on a diurnal scale

Continuous change in the scope of horizontal heat impact formed the horizontal heat impact cycle (HHIC), which varied with the weather and seasonal conditions on a diurnal scale. According to our calculated results along the CSMGT, there were three categories of diurnal-scale patterns in the HHIC: Pattern I, Pattern II and Pattern III. Pattern I was a complete cycle, whereas Pattern II and Pattern III exhibited incomplete cycles.

**Figure 6:**
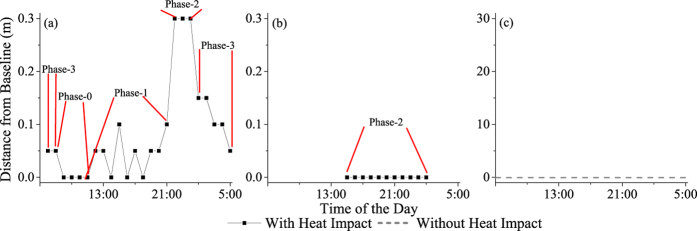
Different patterns in the horizontal heat impact cycle. (**a**) Pattern I; (**b**) Pattern II; (**c**) Pattern III.

Pattern I: based on the temporal and spatial variations in horizontal heat impact along the CSMGT, a complete cycle of horizontal heat impact could be divided into four temporal phases on a diurnal scale: Phase-0, Phase-1, Phase-2 and Phase-3, as shown in Fig. 6.

Phase-0: the spatial distribution of soil temperatures exhibited no gradients along the CSMGT, and the soil temperature could have increased or decreased at any time. This phase usually appeared at dawn and in the morning (Fig. 6a).

Phase-1: this phase usually appeared between sunrise and sunset. Soil temperatures increased and then decreased. The scope of horizontal heat impact gradually increased from 0 m to a certain maximum value (Fig. 6a).

Phase-2: this phase usually appeared at nightfall or dawn when the solar radiation was weak or absent. The soil temperatures decreased gradually to a low value. The scope of horizontal heat impact gradually rose and finally remained at a maximum value for a length of time (depending on weather or meteorological conditions) (Fig. 6a).

Phase-3: the scope of horizontal heat impact decreased from a certain value and then gradually disappeared. The soil temperatures and ΔT_0–50_ decreased (Fig. 6a).

These four phases form a complete cycle in the horizontal heat impact cycle and were clearly observed along the CSMGTs on south-facing external walls on sunny days in summer (Fig. 6a).

Pattern II: this type of pattern often lacked several phases. This situation usually appeared in association with south-, east- and west-facing external walls on cloudy days, east- and west-facing external walls on sunny days in winter, and north-facing external walls on sunny days (Fig. 6b).

Pattern III: this type of pattern usually appeared in association with much less of the solar radiation directly entering the buildings, which meant the buildings contributed less heat to the soil of the adjacent green spaces. Therefore, in these situations, the horizontal heat impact on soil was generally absent. This pattern usually appeared in association with north-facing external walls on cloudy days (Fig. 6c).

### Energy mechanism in the soil surface layer of adjacent green space

**Figure 7:**
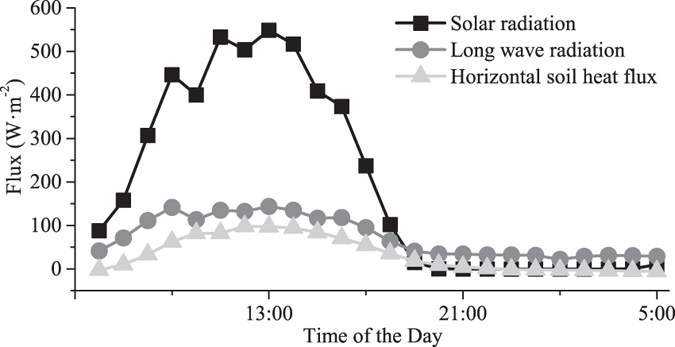
Diurnal changes in solar radiation, long-wave radiation and soil heat fluxes in the CSMGT.

The soil temperature gradient varied with diurnal time, exhibiting a complete process of ecological energy fluxes. T_0_ was driven by different predominant energy factors during a HHIC. Figure 7 shows the diurnal changes in solar radiation, long-wave radiation (solar radiation reduces the net radiation) and horizontal soil heat fluxes at the observation sites. We identified four typical phases of this ecological energy process by considering the key energy factors, such as solar radiation, long-wave radiation and horizontal soil heat fluxes from or into the external walls of buildings, which affected the diurnal dynamics of the soil temperature gradients.

1) Phase-0: changes in the soil temperature were driven by solar radiation and long-wave radiation between the atmosphere and soil. During this phase, the soil temperature showed no regular changes along the CSMGT. Heat fluxes between buildings and the soil did not play a major role in controlling the soil temperature. No stable soil temperature gradients formed in the CSMGT in this phase.

2) Phase-1: the combination of effects of solar radiation, long-wave radiation and heat fluxes dominated the soil temperature in this phase. T_0_ was significantly higher than the soil temperature of other observation points (P < 0.05), temperature differences between different observation points along the CSMGT gradually formed (P < 0.05), and the Stable Point gradually moved farther from the Initial Point, eventually arriving at a stable point as the scope of horizontal heat impacts gradually increased in the CSMGT. This phase mainly appeared at sunset as the heat source changed from solar radiation to the building external walls. Soil temperature gradients clearly formed along the CSMGT in this phase.

3) Phase-2: soil temperatures were changed by long-wave radiation, the heat fluxes entering the atmosphere and the heat fluxes from buildings when the solar radiation decreased to a very low level, which were nearly zero in this phase. The soil temperature decreased, the scope of horizontal heat impacts reached its highest levels and remained stable for a certain length of time (depending on the meteorological conditions). The location of the Stable Point remained unchanged, and the soil temperatures of different observation points between the Initial Point and the Stable Point along the CSMGT differed significantly (P < 0.05). A stable soil temperature gradient formed in the CSMGT in this phase.

4) Phase-3: long-wave radiation determined the soil temperature during this phase. The scope of horizontal heat impacts began to decrease, and the decreasing trend of soil temperature destabilized, although the soil temperatures continued to decrease. The heat fluxes from buildings were weak or even negative because the heat energy stored in buildings was almost exhausted. Finally, air temperature became a dominant factor affecting the soil temperature.

Therefore, the combination of effects of solar radiation, long-wave radiation and heat fluxes among the soil, buildings and atmosphere led to the formation of four different phases in the HHIC. Different meteorological conditions and shading from buildings caused irregular patterns in the soil temperature gradient. The diurnal dynamics of HHIC in the surface soil layer was the result of effects of heat fluxes between buildings and soil and atmospheric energy processes such as solar radiation and long-wave radiation.

### Changes in the soil temperature gradient in different weather conditions and seasons

Changes in the soil temperature gradient (GT) were defined as the soil temperature gradient, a variable of spatial heterogeneity of the soil temperature along the CSMGT, indicating the spatial variation in the soil temperature, with its distance away from the Initial Point along the CSMGT, as follows:


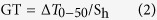


where ΔT_0–50_ is the intensity of horizontal heat impacts by constructions, and S_h_ is the scope of horizontal heat impacts by constructions; usually, the value of GT is expressed by an hour-scale calculation by the above formula (2) in this study. In addition, GT_m_ is defined as the mean value of the soil temperature gradient (GT) on a diurnal scale (24 hours) of the observation interval of time for the CSMGT, at a certain observational site under different weather conditions and seasons, as follows:


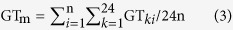


where GT_m_ is mean temperature gradient of the soil, GT_*ki*_ is the per-hour averaging value of the soil temperature gradient, *k* is the *k*th observational hour of a diurnal time scale at the CSMGT of a certain external wall, and n is the number of observational days for each type of diurnal patterns of each external wall along a CSMGT, at a certain observational site under certain weather conditions and seasons (n = 4–18 days in our study). The maximum value of GT is calculated when S_h_ just reaches its maximum, while the minimum value of GT is calculated when S_h_ arrives at its minimum.

As shown in Table 5, for Pattern I, when the value of GT was maximized, the scope of horizontal heat impacts just reached the minimum level of S_h_ (this value dropped between 0.015 m and 0.11 m in our study) and occurred during phase-1 or phase-2. The minimum GT appeared at the end of phase-2 when the scope of horizontal heat impacts began to narrow down or disappear. GT_m_ was larger on sunny days than on cloudy days for the same external wall in the same season. However, there were certain exceptions. In winter, the opposite trend occurred at the west external wall; the maximum value of GT did not appear at the instant the horizontal heat impact reached the minimum value of S_h_ (between 0.015 m and 0.11 m). GT_m_ was larger on cloudy days than on sunny days for the west external wall in winter. For Pattern II, the situation was similar to Pattern I, except that S_h_ changed between 0.007 m and 0.10 m when the GT value was maximized.

### Formation of soil temperature gradient patterns and intensity of horizontal heat impacts of buildings in the CSMGT

Among the factors influencing the soil temperature, the dominant factors that influenced the diurnal and seasonal dynamics of the soil temperature gradients were solar radiation, atmospheric energy processes, soil texture, water content, micro-topography, emissions of anthropological heat and shading by urban structures.

The soil temperature gradient pattern in the CSMGT consisted of the intensity and scope of horizontal heat impacts by buildings (ΔT_0–50_ and S_h_), atmospheric energy processes, and the rate of spatial changes in the horizontal heat impact along the CSMGT.

The heat flow between buildings and soil and the transfer between atmosphere and soil were the two key driving forces that changed soil temperatures in the green space. The process of heat transfer was caused by the interactions among solar radiation, atmospheric energy processes and shading due to structures. Shading by urban structures cools the soil, whereas solar radiation, emissions of anthropological heat and atmospheric energy entering the soil warm the soil. Therefore, the formation of soil temperature gradient patterns in the CSMGT could be determined by these two types of processes based on a relationship of positive and negative feedback.

From an energy balance perspective, the interrelations among different energy flows between the atmosphere, soil and urban structures dominated the soil temperature gradient patterns in the CSMGT, and these energy processes exerted direct and indirect influences on the dynamics of the patterns. The direct atmospheric fluxes of energy entering the soil weakened the pattern, whereas emissions of anthropological heat from urban citizens’ activities and structures intensified the pattern. Solar radiation directly influenced the heat storage and heat fluxes of buildings, which further positively affected the soil temperature by generating heat fluxes from the buildings to the soil.

### Influencing mechanisms of typical micrometeorological factors on the soil temperature for different external walls

External walls of different orientations have different sunshine durations, demonstrating different energetic inputs and other micrometeorological conditions for both soil and constructions. Therefore, Different external walls produce different dynamics in the soil temperature and gradient patterns of it, which are dominantly influenced by different weather conditions, seasons, anthropological heat and urban structures. Several characteristics are described as follows:

1. Geometry and orientation of structures: the building geometry and orientation of different external walls created diverse micro-meteorological environments (for example, via the shading effect) and influenced changes in soil temperatures in different weather conditions and seasons. For example, different patterns of solar radiation and shading conditions were associated with different external wall orientations, causing differences in the quantity of radiant energy and leading to different soil temperature gradient patterns for the different external walls (Fig. 2A4, B4, a4 and b4).
2. Diurnal and seasonal patterns of micro-meteorological factors: when the diurnal and seasonal changes in the micro-meteorological factors along the CSMGT were stable, the soil temperatures also became regular at diurnal and seasonal scales. Therefore, the changes in micro-meteorological conditions are the primary driving forces that determined the dynamics of the soil temperature gradient patterns (Fig. 2A4 and b4).
3. Energy exchange between the atmosphere, soil and buildings: among many micro-meteorological factors, energy exchange (including heat) had a unique contribution to the soil temperature changes. For example, the solar radiation and mean air temperature were the highest in summer and the lowest in winter. The difference in soil temperature between the Initial Point and the Stable Point was caused by the interactions among different energy fluxes, including long-wave and short-wave radiation (e.g., solar radiation), between the soil, atmosphere and buildings. These interactions dominated the changes in heat fluxes from the atmosphere or buildings to the soil and the *vice versa*, especially at night. These interactions explain the different T_0_, T_50_ and ΔT_0–50_ values (Fig. 2A1 to A4 and Fig. 2a1 to a4).

## Methods

### Site conditions

Description of the location: the research site was located in Beijing’s Haidian District (in the northern temperate zone, between 115.7°E-117.4°E and 39.4°N-41.6°N, Fig. 1a), an area with urban structures and loamy soil. Figure 1b,c provides a map of the locations of the CSMGT observation sites.

Description of the climate: the area features a rather dry, monsoon-influenced humid continental climate with a hot and humid summer and a cold and dry winter. The solar radiation is on average 4.69 × 10^6^–5.70 × 10^6^ kJ·m^−2^. The durations of sunshine for different seasons in Beijing are as follows: 240–260 hours per month in spring, 230–245 hours per month in autumn, 230 hours per month in summer, and 170–190 hours per month in winter. Precipitation is unequally distributed throughout the year, and 80% of the precipitation falls during summer.

Description of soil thermal properties: in the study area, the soil thermal conductivity changed between 0.36 W·m^−1^·K^−1^ and 1.51 W·m^−1^·K^−1^, the soil specific heat capacity changed between 847.16 J·kg^−1^·K^−1^ and 1098.94 J·kg^−1^·K^−1^, the density of soil changed between 1325.33 kg·m^−3^ and 1427.50 kg·m^−3^, and the soil thermal diffusivity changed between 2.9 × 10^−7^  m^2^·s^−1^ and 1.3 × 10^−6^  m^2^·s^−1^. All parameters were investigated as section S1.

Description of the land cover: the land cover varied seasonally at the observation sites. All of the trees were under the height of 4 m in the observation sites. The vegetation cover was homogeneous and sunshine was not blocked by vegetation at the observation sites. Air conditions did not influence the soil temperature, due to absence or being used for every observation transect. Radiation reflected by external walls was not considered in this research. This study focused on the surface soil layer where energy exchange is active, hence the situations of deep soil layer was not concerned either. Detailed descriptions of the observation sites are provided in Table S2 in the Supplementary Information.

Description of the constructions: most of constructions in Beijing city are square, rectangular-shape or the combination of the two types of forms that are very convenient for doing field observations from east, south, west and north wall sides. Therefore, external walls with four types of orientations were chosen to ensure enough statistical sets of data for horizontal heat impact examination. In the study area, wall foundations are greater than 3 m and may influence the temperature of deep soil layer. However, wall foundations are not the primary factor to influence the temperature of surface soil layer (at the depth of 0–0.025 m in this research).

### Layout of the CSMGT

The CSMGT has been developed and used in this research. The gradient transect analysis originated from the method used in Robert. H. Whitaker’s research^30^. Other scholars successfully used Whitaker’s method and acquired abundant results^31^,^32^,^33^. However, Whitaker’s method was previously employed to explore ecological issues on large or moderate scales; until now, no research has revealed whether this method can be suitably applied to smaller scales. We attempted in this study to examine and test this ecological method’s applicability by scaling the process down to the observation level of a metre or centimetre, and we successfully established a micro-gradient transect with an anthropological-ecological interface. Green space soils adjacent to the external walls of buildings were selected as research sites to establish the experimental CSMGT. Soil temperature sensors were installed at different observation points along the CSMGT. The layout of a CSMGT is illustrated in Fig. 1c and is described below:

1) The contact between the building baseline and soil were considered the starting points and named “the Initial Point” (0 m from the building baseline).

2) For one CSMGT, the soil temperature sensors (Figure S5a) were arranged at observation points in the soil surface layer at a depth of 0–0.025 m (Figure S5b). These points were arranged in a straight line, perpendicular to one building’s external wall or in a zigzagging pattern away from the wall. Both patterns began at the Initial Point.

3) At a certain distance from the Initial Points (the edge of the building baseline), the heat transfer from the buildings was too weak to affect the soil temperature (shown in section S3 in the Supplementary Information). The observation points at or beyond this distance were not affected by heat transfer and were considered reference points. The nearest point to the Initial Point was located via statistical analysis of observation data and was named “the Stable Point”.

As described in section S3 of the Supplementary Information, the maximum scope of the horizontal heat impact of buildings on the soil was usually within 0.3 m. Therefore, the observation points along the CSMGT were installed at distances of 0, 0.05, 0.10, 0.15, 0.20, 0.30 and 0.50 m from the Initial Point. The observation point of 0.50 m was considered control or reference.

### *In situ* observations

*In situ* observations are widely used in ecological studies as reliable methods for data generation^34^,^35^,^36^,^37^. *In situ* measurements were used to investigate soil temperatures in this research. Based on the results of the statistical analyses in sections S4 and S5 of the Supplementary Information in this study, urban structures with different geometric configurations and material textures have similar patterns of horizontal heat impact on the temperature of the surface soil layer under the same weather and season conditions. Thus, the observation results from a CSMGT at any external wall were sufficiently representative to estimate the actual horizontal heat impact on the temperature of the surface soil layer for all similar external walls under the same or similar meteorological conditions.

### Data acquisition

Meteorological data were recorded at a small weather station, which featured an observation interval of 60 seconds and a logging interval of 10 minutes, including air temperature (accuracy of sensor: 0.25 K), relative humidity (accuracy of sensor: 2%), solar radiation (accuracy of sensor: 3%), net radiation (sensitivity of sensor: 10 μV·W^−1^·m^−2^) and the horizontal heat flux between construction and soil (accuracy of sensor: 5%). The sensors for air temperature, relative humidity solar radiation and net radiation were set at 2 m above the ground surface (Figure S6a). The soil heat flux plate was just set beneath the ground surface, at the construction baseline, with the top facing the construction and the bottom facing the soil (Figure S6b).

The soil temperatures of the green spaces were observed using temperature sensors (with a 0.2 K accuracy) and recorded by data loggers. The observation interval was 30 seconds, and the logging interval was 1 minute. The obtained data were averaged every 10 minutes. Thus, 6 numerical values for every soil temperature sensor were acquired each hour and processed as an observation group for per hour (0–59^th^ minute).

### Data processing

The observation groups for all observation points in the CSMGT were analysed statistically by one-way ANOVA (Duncan method, with a confidence interval of 95%) to calculate significant differences between observation groups. The data sets of this research assumed a good normal distribution. As shown in Table S5, a small part of our tests of normality were listed randomly (due to the great plenty of data, only part of them were listed), all the Sig values are greater than 0.05. Thus, the data sets showed a good normal distribution.

The relationship between the soil temperature difference between T_0_ and T_50_ and micrometeorological factors was analysed by stepwise regression, which is widely used in a large variety of environmental applications^38^. Independent variables were selected or rejected in the stepwise regression based only on the condition that the significance is less than 0.05 or greater than 0.1.

Both one-way ANOVA and stepwise regression were calculated using SPSS (Statistical Package for the Social Sciences) statistical software version 17.0.

### Calculation of the scope of horizontal heat impact

Theoretically, constructions are heat sources to the adjacent soil and the soil temperature decreases from the Initial Point. Using one-way ANOVA, a significant difference between observation points, including two adjacent points, could be identified every hour. Significant differences between two adjacent observation points could be used to identify whether there is horizontal heat impact.

1) In situations with significant differences between two adjacent points within one hour, the building is exerting a horizontal heat impact on the adjacent green space soil, and a soil temperature gradient appears in the CSMGT (P < 0.05). If there is no significant difference between two adjacent points (P > 0.05), no horizontal heat impacts on the soil or soil temperature gradients are present. The data from the points without significant differences are considered candidate reference or stable points for the soil temperature. Due to homogeneous grass cover, vegetation shading did not influence this research. The nearest reference point in the CSMGT to the Initial Point is termed “the Stable Point” (Fig. 1c), and all points behind “the Stable Point” are likewise considered as Stable Points.

2) If the back observation point statistically shows a higher temperature between any two adjacent observation points or the temperature difference between two adjacent observation points in the CSMGT is less than 0.2 K (the accuracy of the soil temperature sensor), the data indicate that there are no horizontal heat impacts on soil or soil temperature gradients between the two adjacent observation points.

## Additional Information

**How to cite this article**: Zhou, H. *et al.* Horizontal Heat Impact of Urban Structures on the Surface Soil Layer and Its Diurnal Patterns under Different Micrometeorological Conditions. *Sci. Rep.* **6**, 18790; doi: 10.1038/srep18790 (2016).

## Supplementary Material

Supplementary Information

## Figures and Tables

**Table 1:** Diurnal changes in T_0_, T_50_ and ΔT_0–50_.

Site	Season	Weather	T_0_						T_50_					ΔT_0–50_				
			T_max_ (K)	t_Tmax_	T_min_ (K)	t_Tmin_	Duration of Heat Source	T_m_ (K)	T_max_ (K)	t_Tmax_	T_min_ (K)	t_Tmin_	T_m_ (K)	ΔT_max_ (K)	t_ΔTmax_	ΔT_min_ (K)	t_ΔTmin_	ΔT_m_ (K)
South 1	Autumn	Sunny	302.50	13:00	290.61	6:00	6:00–5:00	294.73	298.04	13:00	289.81	6:00	292.82	4.92	14:00	0.80	6:00	1.91
		Cloudy	299.25	14:00	289.75	5:00	6:00–5:00	293.19	296.64	14:00	289.27	5:00	292.38	2.62	14:00	0.38	9:00	0.81
South 5	Winter	Sunny	281.96	14:00	273.50	7:00	6:00–7:00 & 9:00–5:00	276.73	280.44	14:00	273.49	7:00	276.02	1.80	16:00	−0.01	8:00	0.71
		Cloudy	279.66	15:00	274.16	7:00	6:00–5:00	276.25	278.63	14:00	273.93	7:00	275.67	1.24	16:00	0.19	9:00	0.58
South 1	Spring	Sunny	305.85	14:00	289.85	6:00	6:00–5:00	296.65	303.90	12:00	288.94	6:00	295.38	2.45	14:00	0.16	9:00	1.27
		Cloudy	296.03	13:00	289.50	5:00	6:00–5:00	292.48	295.31	13:00	288.71	5:00	291.56	1.18	19:00	0.72	13:00	0.92
South 1	Summer	Sunny	308.55	13:00	295.83	6:00	6:00–5:00	301.61	307.03	13:00	294.22	6:00	299.23	3.38	18:00	1.24	10:00	2.38
		Cloudy	301.54	13:00	296.18	5:00	6:00–5:00	298.60	299.83	12:00	294.91	5:00	296.80	2.40	17:00	1.28	5:00	1.80
North 2	Autumn	Sunny	285.59	16:00	282.79	7:00	6:00–5:00	284.14	284.89	15:00	282.19	6:00	283.42	1.02	20:00	0.39	12:00	0.72
		Cloudy	286.64	16:00	283.48	7:00	6:00–5:00	284.93	286.28	15:00	282.94	6:00	284.50	0.72	18:00	0.04	12:00	0.43
North 2	Winter	Sunny	273.14	15:00	271.26	7:00	13:00–17:00	272.01	272.89	15:00	271.79	5:00	272.26	0.26	13:00	−0.55	8:00	−0.25
		Cloudy	273.00	15:00	270.92	7:00	13:00–20:00	271.99	272.76	15:00	271.56	7:00	272.17	0.24	15:00	−0.64	7:00	−0.18
North 6	Spring	Sunny	290.68	16:00	286.13	6:00	6:00–5:00	288.38	290.09	16:00	285.76	6:00	287.73	1.34	20:00	0.05	10:00	0.65
		Cloudy	290.98	16:00	286.63	5:00	6:00–5:00	288.79	290.51	16:00	286.15	5:00	288.20	1.19	19:00	0.16	13:00	0.59
North 2	Summer	Sunny	300.40	16:00	293.61	6:00	6:00–5:00	296.32	299.69	16:00	293.08	6:00	295.50	1.57	18:00	−0.18	9:00	0.82
		Cloudy	299.63	13:00	294.81	5:00	6:00–5:00	297.25	298.37	13:00	294.21	5:00	296.21	1.46	17:00	0.60	5:00	1.03
East 3	Autumn	Sunny	292.02	11:00	288.52	7:00	6:00–5:00	290.33	290.35	11:00	287.45	7:00	288.71	2.05	16:00	1.05	8:00	1.62
		Cloudy	290.05	14:00	288.98	5:00	6:00–5:00	289.65	288.86	14:00	288.32	5:00	288.62	1.37	6:00	0.66	5:00	1.03
East 3	Winter	Sunny	273.56	15:00	271.90	8:00	6:00–9:00 & 11:00–5:00	273.00	273.46	10:00	270.86	7:00	272.19	1.07	22:00	−0.08	10:00	0.81
		Cloudy	—	—	—	—	—	—	—	—	—	—	—	—	—	—	—	—
East 3	Spring	Sunny	287.25	12:00	276.45	7:00	6:00–5:00	280.72	286.81	12:00	274.81	6:00	278.98	2.22	14:00	0.44	12:00	1.74
		Cloudy	278.83	14:00	277.18	5:00	6:00–5:00	277.98	277.70	13:00	275.85	6:00	276.65	1.71	6:00	1.07	12:00	1.33
East 7	Summer	Sunny	306.60	14:00	297.27	6:00	6:00–5:00	301.09	303.97	13:00	296.56	5:00	299.24	3.90	16:00	0.22	10:00	1.85
		Cloudy	301.88	15:00	297.27	5:00	6:00–5:00	299.49	301.04	13:00	296.86	5:00	298.62	1.65	18:00	0.12	12:00	0.88
West 4	Autumn	Sunny	301.28	14:00	285.62	6:00	6:00–11:00 & 13:00–5:00	290.98	298.93	14:00	284.48	6:00	289.47	3.76	15:00	−0.49	12:00	1.51
		Cloudy	296.36	14:00	288.08	7:00	6:00–11:00 & 13:00–5:00	291.23	295.63	14:00	287.18	6:00	290.38	1.25	18:00	−0.13	12:00	0.85
West 8	Winter	Sunny	278.69	15:00	275.02	7:00	6:00–13:00 & 16:00–5:00	276.29	279.41	14:00	273.31	7:00	275.24	1.72	6:00	−1.15	14:00	1.05
		Cloudy	281.34	15:00	276.35	7:00	6:00–9:00 & 17:00–5:00	278.18	282.99	15:00	274.11	5:00	277.43	2.43	5:00	−2.06	14:00	0.75
West 8	Spring	Sunny	285.88	15:00	277.13	7:00	6:00–13:00 & 16:00–5:00	279.78	286.55	15:00	275.07	7:00	278.57	2.15	5:00	−1.30	14:00	1.22
		Cloudy	279.79	13:00	277.82	5:00	6:00–5:00	278.77	278.70	13:00	276.00	6:00	277.35	2.16	6:00	1.09	13:00	1.42
West 4	Summer	Sunny	299.72	14:00	295.03	6:00	6:00–11:00 & 15:00–5:00	296.77	301.02	13:00	292.91	6:00	295.19	2.31	4:00	−2.03	13:00	1.58
		Cloudy	297.37	13:00	294.04	5:00	6:00–5:00	295.83	296.51	13:00	292.13	5:00	293.97	2.43	6:00	0.86	13:00	1.86

Note: T_max_ is the highest temperature per 24 hours, t_Tmax_ is the time of the appearance of the highest temperature, T_min_ is the lowest temperature per 24 hours, t_Tmin_ is the time of the appearance of the lowest temperature, and T_m_ is the average soil temperature at the diurnal scale. ΔT_max_ is the difference between the maximum temperatures of the Initial Point and the Stable Point, t_ΔTmax_ is the difference in the timing of the appearance of maximum temperatures between the Initial Point and the Stable Point, ΔT_min_ is the difference between the minimum temperatures of the Initial Point and the Stable Point, t_ΔTmin_ is the difference in the timing of the appearance of the minimum temperatures of the Initial Point and the Stable Point. ΔT_m_ is the difference in the average soil temperatures between the Initial Point and the Stable Point on a diurnal scale. The symbol “—” denotes “not applicable”.

**Table 2:** Maximum S_h_ and total temporal duration of impacts for different side walls in various weather and seasonal conditions.

Site	Weather	Autumn		Winter		Spring		Summer	
		S_h- max_ (m)	Dur_total_ (h)	S_h- max_ (m)	Dur_total_ (h)	S_h- max_ (m)	Dur_total_ (h)	S_h- max_ (m)	Dur_total_ (h)
South	Sunny	0.15	19	0.10	8	0.10	11	0.30	21
	Cloudy	0.15	9	0.10	6	0	10	0.05	5
North	Sunny	0	11	0	5	0.15	16	0.05	21
	Cloudy	None	None	None	None	None	None	0.05	12
East	Sunny	0.20	23	0.05	10	0.10	19	0.20	14
	Cloudy	0	16	—	—	0.05	15	0.05	13
West	Sunny	0.10	21	0.15	20	0.10	19	0.20	20
	Cloudy	0.05	11	0.10	22	0.05	16	0.15	23

Note: S_h- max_ is the maximum value of horizontal heat impact scope.

**Table 3:** Difference in the mean soil temperature (ΔMT_0–50_) between the Initial Point and the Stable Point and direction of the heat flows.

Location	Autumn		Winter		Spring		Summer	
	ΔMT_0–50_ (K)	Direction of heat flow	ΔMT_0–50_ (K)	Direction of heat flow	ΔMT_0–50_ (K)	Direction of heat flow	ΔMT_0–50_ (K)	Direction of heat flow
South	1.61	T_0_ → T_50_	0.68	T_0_ → T_50_	1.23	T_0_ → T_50_	2.18	T_0_ → T_50_
North	0.65	T_0_ → T_50_	−0.23	T_50_ → T_0_	0.64	T_0_ → T_50_	1.09	T_0_ → T_50_
East	1.36	T_0_ → T_50_	0.81	T_0_ → T_50_	1.51	T_0_ → T_50_	1.68	T_0_ → T_50_
West	0.84	T_0_ → T_50_	1.00	T_0_ → T_50_	1.14	T_0_ → T_50_	1.34	T_0_ → T_50_

**Table 4:** Regression model coefficients.

	Unstandardized Coefficients		Standardized Coefficients	Sig.
	b	Std. Error	Beta	
(Constant)	2.126	0.270		<0.0001
RH	−0.032	0.001	−0.438	<0.0001
SR	−5.101	0.087	−0.827	<0.0001
HHF	0.056	0.002	0.726	<0.0001
WS	0.233	0.045	0.061	<0.0001
DW	8.982	1.254	0.084	<0.0001
AT	0.052	0.008	0.147	<0.0001

Note: RH is relative humidity, SR is solar radiation, HHF is horizontal heat flux, WS is wind speed, DW is soil moisture difference and AT is air temperature.

**Table 5:** GT values among three categories of HHIC patterns at a typical diurnal scale for different seasons.

Pattern	Season	Side	GT in Sunny Day (K/m)				GT in Cloudy Day (K/m)			
			Max	Min	GT_m_	GT*	Max	Min	GT_m_	GT*
Pattern I	Autumn	South	—	—	—	—	—	—	—	—
		North	—	—	—	—	—	—	—	—
		East	38.37	15.21	24.84	15.30	—	—	—	—
		West	75.26	15.04	31.51	19.52	24.55	13.20	21.30	24.55
	Winter	South	—	—	—	—	18.36	12.43	16.34	12.43
		North	14.45	12.16	13.57	14.10	—	—	—	—
		East	21.07	20.47	20.68	20.47	—	—	—	—
		West	25.60	10.20	15.14	13.30	—	—	—	—
	Spring	South	62.57	15.17	31.80	37.41	17.25	15.21	16.35	16.28
		North	17.78	7.19	11.93	11.27	30.28	8.22	15.25	10.71
		East	33.69	17.26	27.87	30.08	—	—	—	—
		West	45.11	13.29	30.29	23.51	—	—	—	—
	Summer	South	62.31	13.71	28.19	16.28	36.88	7.53	21.60	7.65
		North	28.12	12.85	18.81	28.12	—	—	—	—
		East	—	—	—	—	—	—	—	—
		West	24.19	7.35	19.48	18.28	23.68	8.25	16.28	8.25
Pattern II	Autumn	South	110.32	6.55	18.78	15.70	6.88	6.03	6.45	6.88
		North	22.84	11.84	17.57	17.94	—	—	—	—
		East	—	—	—	—	—	—	—	—
		West	—	—	—	—	—	—	—	—
	Winter	South	29.44	13.02	18.65	13.02	—	—	—	—
		North	—	—	—	—	—	—	—	—
		East	21.16	18.79	20.16	18.79	—	—	—	—
		West	35.98	6.27	17.10	13.58	48.62	35.50	42.06	48.62
	Spring	South	40.78	16.65	23.45	16.65	30.86	14.54	19.36	15.86
		North	34.46	16.78	29.13	16.78	—	—	—	—
		East	—	—	—	—	—	—	—	—
		West	42.34	19.47	33.96	31.49	43.21	12.98	26.65	14.82
	Summer	South	62.91	31.69	46.54	61.14	50.16	25.15	38.05	38.84
		North	37.91	14.05	21.65	24.68	28.46	17.15	23.44	28.46
		East	38.21	10.05	20.84	14.85	22.95	0.97	15.18	0.97
		West	23.25	9.61	19.88	21.35	22.71	13.60	17.09	13.60

Note: Max is the maximum of GT, Min is the minimum of GT, GT_m_ is the mean value of GT at a diurnal scale, and “*” is a value of GT when the scope of horizontal heat impact maximizes. When there was no horizontal heat impact only occurred in Pattern III, and its value was equal to zero in Pattern II as well as the indicator of S_h_ was applicable for some situations in Pattern I and II, the values of GT were then denoted as “—”.
