# Supplementary material for: Horizontal Heat Impact of Urban Structures on the Surface Soil Layer and Its Diurnal Patterns under Different Micrometeorological Conditions
Source: Sci Rep. 2016 Jan 5;6:18790. doi: 10.1038/srep18790 (PMC4700467; doi:10.1038/srep18790)
Supplement: Supplementary Information [file srep18790-s1.pdf]

Horizontal Heat Impact of Urban Structures on the Surface Soil Layer and Its Diurnal Patterns under Different Micrometeorological Conditions

Hongxuan Zhou[1], Dan Hu[1,*], Xiaolin Wang[1], Fengsen Han[1], Yuanzheng Li[1], Xiaogang Wu[2,3], Shengli Ma[4]

[1] State Key Laboratory of Urban and Regional Ecology, Research Center for Eco-Environmental Sciences, Chinese Academy of Sciences, Beijing 100085, PR China

[2] College of Urban and Rural Construction, Shanxi Agricultural University, Taigu County, Shanxi Province, 030801, PR China

[3] Environment and Sustainability Institute, University of Exeter, Penryn, Cornwall TR10 9FE, U.K.

[4] College of Eco-Environmental Engineering, Qinghai University, Xining, Qinghai, 810016, PR China

Supplementary Information

S1 Measurement of soil thermal properties.

Using a thermal properties analyzer (the sensor accuracy of the thermal conductivity is 5%) to measure soil thermal conductivity at the eight chosen site. Soil specific heat capacity was measured by applying the method of mixing calorimetry [1]. Density of soil was investigated by a precision electronic balance and measuring the radius of soil sampler and the length of the soil sample. The soil thermal diffusivity was calculated by the equation (S1).

$$\alpha=\frac{\lambda}{C\times\rho} \quad \text{(S1)}$$

Where α is the soil thermal diffusivity ($m^2\ s^{-1}$), λ is the soil thermal conductivity ($W\cdot m^{-1}\ K^{-1}$), C is the soil specific heat capacity ($J\ kg^{-1}\ K^{-1}$), and ρ is the density of soil ($kg\cdot m^{-3}$). All thermal parameters of soil in the study area are exhibited in Table S1.

Table S1 | Soil thermal properties.

Side wall	Number of sites	λ ($W\ m^{-1}\ K^{-1}$)	C ($J\ kg^{-1}\ K^{-1}$)	ρ ($kg\ m^{-3}$)	α ($m^2\ s^{-1}$)
South	1	1.51	847.16	1363.81	1.30×10^{-6}
North	2	0.95	938.71	1402.86	7.24×10^{-7}
East	3	1.12	910.98	1336.67	9.20×10^{-7}
West	4	0.82	1098.94	1363.33	5.47×10^{-7}
South	5	1.12	1020.67	1375.20	7.98×10^{-7}
North	6	1.31	895.54	1427.50	1.03×10^{-6}
East	7	1.28	1064.04	1325.33	9.10×10^{-7}
West	8	0.36	905.64	1378.33	2.88×10^{-7}

S2 Observation site information.

Table S2 describes the observation sites in this research, including the sizes of the buildings and green space, types of land cover, atmospheric temperature and relative humidity. The atmospheric temperature and relative humidity were acquired from weather station (set by ourselves). Data on the duration of sunshine was calculated using the architecture software Ecotect based on observed meteorological data.

Table S2 | Details of the observation sites in this research.

Season	Side wall	Number of Sites	Construction Size			Green Space Condition				Meteorological Condition		
			L (m)	W (m)	H (m)	Land Cover	L (m)	W (m)	Duration of Sunshine	Whether	AT (K)	RH (%)
Autumn	South	1	66	18	21	G	61	21	8:30~16:00	Sunny	283~298	18~90
										Cloudy	285~295	44~93
	North	2	48	12	18	G	61	21	16:00~17:30	Sunny	277~293	15~82
										Cloudy	278~291	25~86
	East	3	56	10~28	28	G	56	2	9:30~11:30	Sunny	282~296	25~86
										Cloudy	287~289	58~81
	West	4	54	16	40	G	16	4	11:30~16:30	Sunny	282~298	14~84
										Cloudy	284~297	38~87
Winter	South	5	18	62	32	G & DL	25	63	9:15~17:00	Sunny	269~284	8~73
										Cloudy	271~282	11~78
	North	2	48	12	18	G & DL	61	21	None	Sunny	268~279	17~60
										Cloudy	268~278	16~50
	East	3	56	10~28	28	DL	56	2	9:30~11:45	Sunny	269~280	11~28
										Cloudy	-	-
	West	8	54	16	40	DL & BS	16	4	13:30~16:30	Sunny	269~282	11~77
										Cloudy	273~284	14~38
Spring	South	1	66	18	21	G & DL	61	21	6:00~8:00& 9:30~18:15	Sunny	282~299	13~79
										Cloudy	282~295	11~81
	North	6	70	14	18	G & DL	70	14	16:45~18:15	Sunny	282~299	13~74
										Cloudy	282~297	27~74
	East	3	56	10~28	28	DL	56	2	9:30~12:00	Sunny	271~285	8~74
										Cloudy	274~277	34~72
	West	8	54	16	40	DL & BS	16	4	11:30~16:30	Sunny	271~285	8~74
										Cloudy	274~277	34~72
Summer	South	1	66	18	21	G	61	21	8:00~18:00	Sunny	291~308	14~89
										Cloudy	290~307	35~88
	North	2	48	12	18	G	61	21	16:00~19:15	Sunny	290~305	24~89
										Cloudy	291~300	52~89
	East	7	18	62	32	G	25	63	5:30~13:30	Sunny	294~308	11~73
										Cloudy	296~305	41~74
	West	4	54	16	40	G	16	4	11:30~16:30	Sunny	290~308	18~89
										Cloudy	290~303	30~89

Note: L is length, W is width, H is height, AT is atmospheric temperature, RH is relative humidity, G is grass land, DL is dead leaves, BS is bare soil, and "-" is no data.

S3 Quantitative determination of the scope of horizontal heat impact.

According to the literature, on a diurnal scale, soil temperatures change across a vertical depth of 0-0.4 m and in rare situations 0-0.6 m [2]. In certain situations, such as experiments involving the heating of soil, soil temperature changes occurred to a length of 0.3 m, and soil temperatures behind a length of 0.3 m were stable [3]. Until now, no researchers have focused on the horizontal heat impact of buildings on soil temperature. Therefore, the CSMGT method, described in **Methods**, was used to explore the issue in this study. Six observation or experimental transects were arranged in the soil of

green spaces adjacent to the studied buildings. The soil temperatures were recorded hourly and were analysed as described in **Methods**. The results showed that the scopes of horizontal heat impact universally fluctuated within a spatial distance of 0.3 m from the Initial Point in the CSMGT. This pattern was similar to the results of the soil heating experiments [3]. Therefore, the fluctuation in the scope of horizontal heat impacts could be determined within a horizontal distance of 0.3 m from the Initial Point in the CSMGT. See Figure S1 for the results of this analysis.

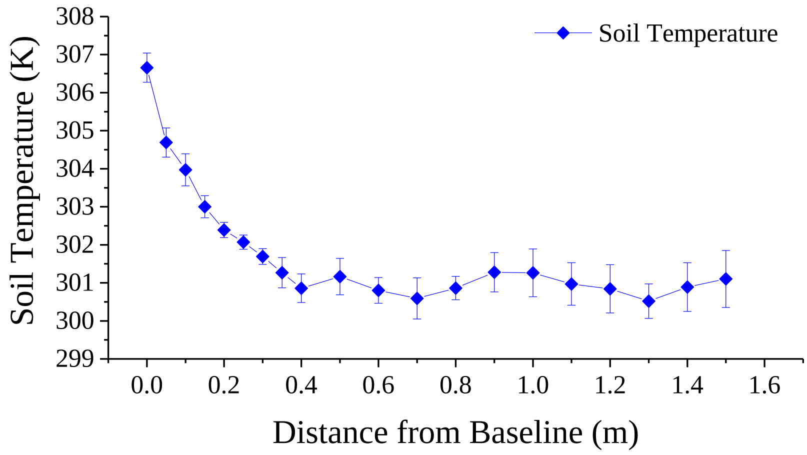

Figure S1 | Observed values for the scope of horizontal heat impact for urban buildings from the Initial Point in the CSMGT.

S4 The scope of horizontal heat impact for buildings with different configurations.

There are a large number of structures with different types of configurations within urban areas, including high-rise buildings, low buildings, flat houses, parking lots, roads, pipelines, etc. The thermal properties, such as heat storage, accumulation and thermal conductivity, for these structures differ. The method mentioned in S3 was also utilized to investigate the spatial distribution of soil temperatures for four categories of building configurations and other urban structures: a high-rise building, a low building, a parking lot and small-scale pavement. The detailed observation results for the buildings and paved surfaces are shown in Table S3.

Table S3 | Attributes of different categories of buildings and urban structures.

Category	Material	Width (m)	Length (m)	Height (m)	Floor Space(m^2)
High-rise Building	Concrete	34	34	69	1167
Low Building	Concrete	66	18	20	1140
Park Lot	Asphalt	31	254	-	7500
Small Pavements	Concrete Brick	5	26	-	133

The horizontal heat impacts of buildings with different configurations on soil temperature in sunny days in autumn and winter were observed and recorded in our research, and the results are shown in Figure S2.

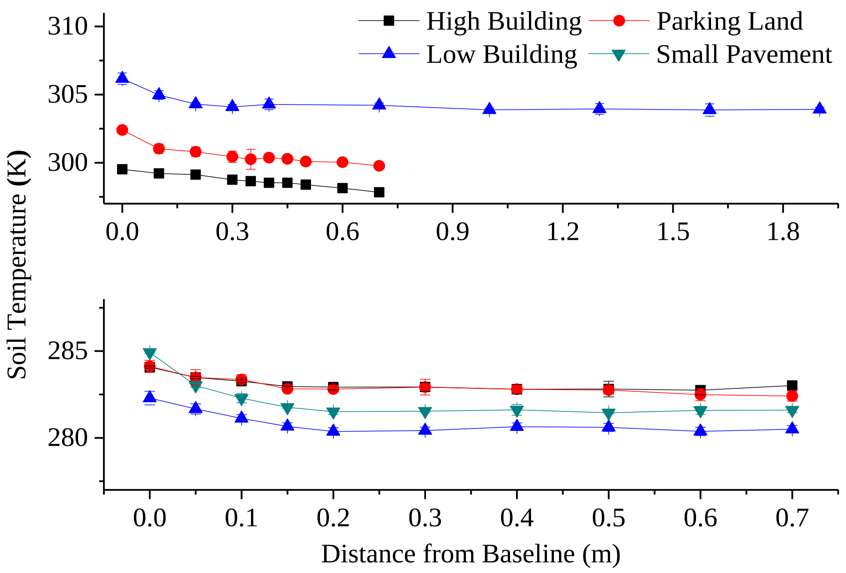

Figure S2 | Scopes of horizontal heat impact for buildings with different configurations on a sunny day in autumn and winter.

For a given period of sunny days in autumn, the soil temperatures of the green space adjacent to three categories of urban structures with different configurations were investigated and analysed. Similar decreasing trends appeared in the CSMGT. The statistical analysis indicated that the soil temperatures were significantly different in the 0.2 m of the CSMGT adjacent to the structures. Therefore, the scopes of horizontal heat impact for different categories of buildings or structures all decreased within a horizontal distance of 0.2 m from the Initial Point. Due to a difference in dates of observation, the actual values of soil temperature differed for each CSMGT. In winter, another

structure was added to the observation sites, but similar results were also obtained; the only disparity was that the scope of the horizontal heat impact decreased to 0.1 m.

On sunny days, the four categories of urban structures with different configurations shared the same scope of horizontal heat impact at the same period of time in autumn and winter. Thus, urban structures with different configurations had the same scope of horizontal heat impact on the soil in the adjacent green space when weather and seasonal conditions were identical.

S5 The scope of horizontal heat impact along observation transect lines parallel to the building baseline.

Two observation transect lines were installed to parallel the building baseline at a distance of 0 m and 0.1 m. Eight observation points were allocated randomly along the parallel lines for a total length of less than or equal to 20 m (Figure S3). The soil temperature of each observation point on the line was investigated three times at the same interval of time of one hour, and recorded as T_0 and T_{10}. The mean soil temperature for each observation point is shown in Figure S4. The results of the statistical analysis indicated that the variations in T_0 and the variations in T_{10} among the observation points along their respective observation transects were not statistically significant ($P>0.05$).

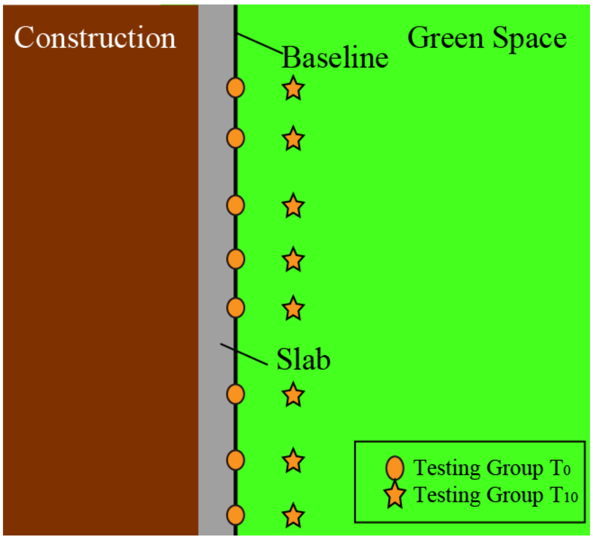

Figure S3 | Layout of the T_0 and T_{10} transects.

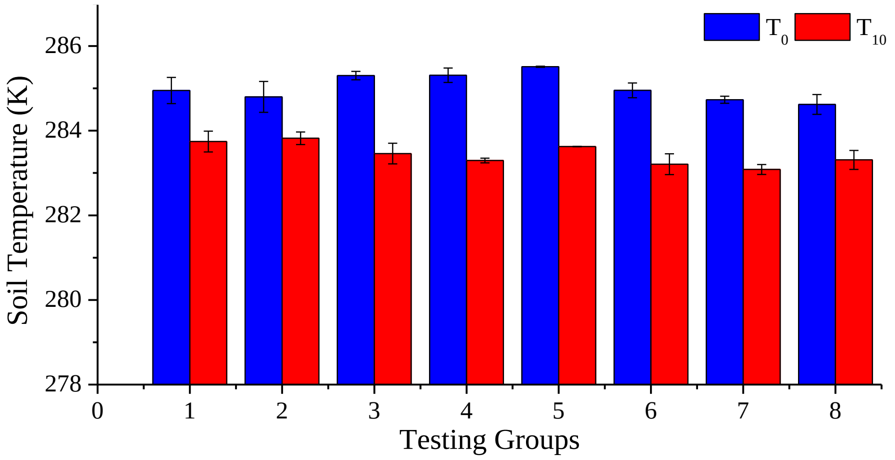

Figure S4 | Scopes of horizontal heat impact along observation transect lines parallel to the building baseline.

Based on these results of analysis, we could conclude that, observation points along observation transect lines parallel to the building baseline were equally influenced by the horizontal heat processes from the buildings. Therefore, soil temperature did not vary among different observation points along any observation transect lines parallel to the building baseline.

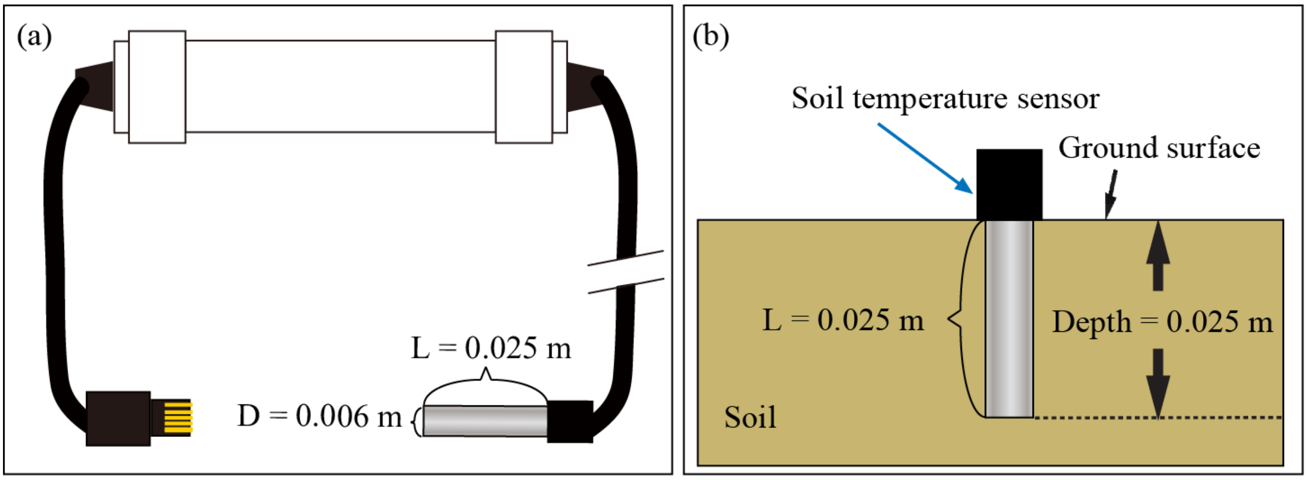

Figure S5 | Soil temperature sensor (a depicts the soil temperature sensor and b shows the arrangement of soil temperature sensor. D means the diameter of soil temperature sensor; L means the length of soil temperature sensor).

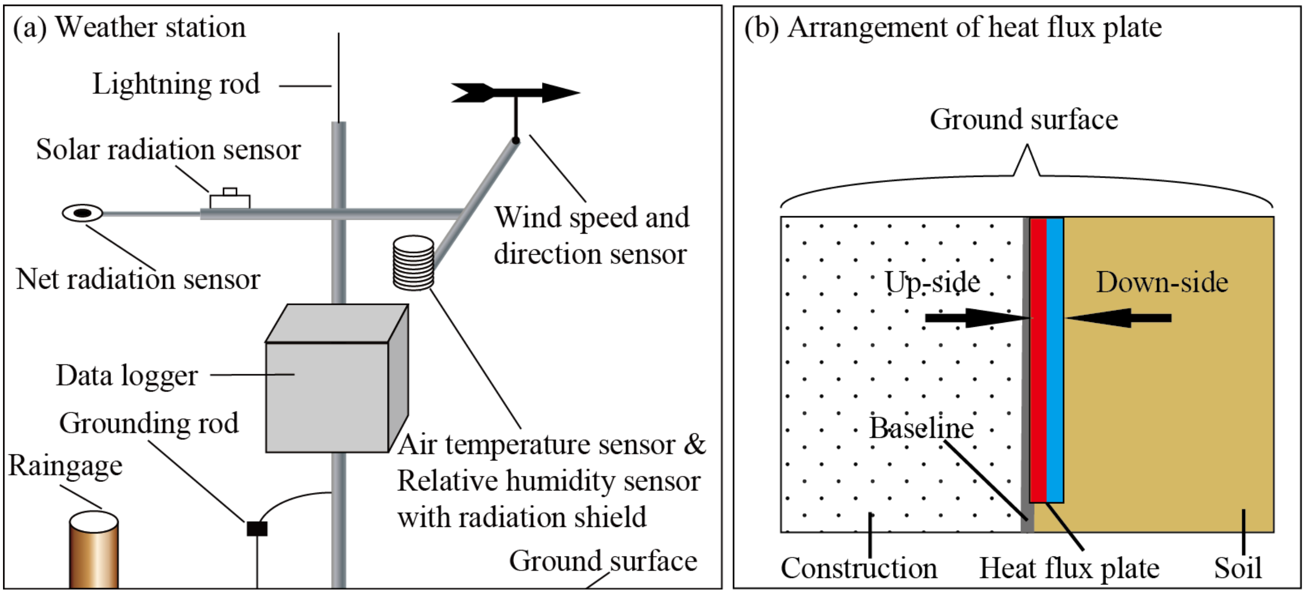

Figure S6 | Arrangement of the weather station and soil heat flux plate.

Table S4 | Observed durations of soil temperatures in the adjacent green space in different seasons.

Location	Durations			
	Autumn	Winter	Spring	Summer
South	2013.9.30-2013.10. 8	2014.1.16-2014.1.28	2014.4.11-2014.4.23	2014.6.20-2014.6.27
North	2013.10.15-2013.10.24	2013.12.21-2013.12.26	2014.4.19-2014.4.27	2014.6.19-2014.6.25
East	2013.10.10-2013.10.13	2013.12.28-2013.12.29	2014.3.1-2014.3.9	2014.8.16-2014.8.26
West	2013.9.25-2013.9.28	2013.12.28-2014.1.5	2014.3.1-2014.3.9	2014.6.1-2014.6.18

Table S5 | Tests of normality.

Season	Time	Orientation	Testing Groups	Sig.
Autumn	12:00	South-facing	T_0	0.931
Autumn	21:00	South-facing	T_{50}	0.096
Autumn	3:00	North-facing	T_{30}	0.990
Autumn	18:00	North-facing	T_0	0.958
Autumn	9:00	East-facing	T_5	0.525
Autumn	15:00	East-facing	T_{20}	0.167
Autumn	6:00	West-facing	T_{10}	0.649
Autumn	0:00	West-facing	T_{15}	0.915
Winter	12:00	South-facing	T_0	0.761
Winter	21:00	South-facing	T_{50}	0.421
Winter	3:00	North-facing	T_{30}	0.373
Winter	18:00	North-facing	T_0	0.076
Winter	9:00	East-facing	T_5	0.536
Winter	15:00	East-facing	T_{20}	0.957
Winter	6:00	West-facing	T_{10}	0.978
Winter	0:00	West-facing	T_{15}	0.710
Spring	12:00	South-facing	T_0	0.259
Spring	21:00	South-facing	T_{50}	0.944

Spring	3:00	North-facing	T_{30}	0.757
Spring	18:00	North-facing	T_0	0.686
Spring	9:00	East-facing	T_5	0.781
Spring	15:00	East-facing	T_{20}	0.909
Spring	6:00	West-facing	T_{10}	0.177
Spring	0:00	West-facing	T_{15}	0.799
Summer	12:00	South-facing	T_0	0.875
Summer	21:00	South-facing	T_{50}	0.930
Summer	3:00	North-facing	T_{30}	0.775
Summer	18:00	North-facing	T_0	0.891
Summer	9:00	East-facing	T_5	0.552
Summer	15:00	East-facing	T_{20}	0.423
Summer	6:00	West-facing	T_{10}	0.184
Summer	0:00	West-facing	T_{15}	0.206

Reference

1 Yu, Z. Measuring the specific heat and thermal conductivity of the soil. *Soils* **1**, 48-51 (1986).

2 Tang, C.-S. *et al.* Urbanization effect on soil temperature in Nanjing, China. *Energy and Buildings* **43**, 3090-3098 (2011).

3 Shao, M. A., Wang, Q. J. & Huang, M. B. *Soil Physics*. (Higher Education Press, 2006).
